# Ergotaminine

**DOI:** 10.1107/S1600536812003674

**Published:** 2012-02-04

**Authors:** Stefan Merkel, Robert Köppen, Matthias Koch, Franziska Emmerling, Irene Nehls

**Affiliations:** a Reference Materials, Department of Analytical Chemistry, BAM Federal Institute for Materials Research and Testing, Richard-Willstätter-Strasse 11, D-12489 Berlin-Adlershof, Germany

## Abstract

The title compound {systematic name: (6a*R*,9*S*)-*N*-[(2*R*,5*S*,10a*S*,10b*S*)-5-benzyl-10b-hy­droxy-2-methyl-3,6-dioxoocta­hydro-8*H*-oxazolo[3,2-*a*]pyrrolo­[2,1-*c*]pyrazin-2-yl]-7-methyl-4,6,6a,7,8,9-hexa­hydro­indolo[4,3-*fg*]quinoline-9-carboxamide}, C_33_H_35_N_5_O_5_, was formed by an epimerization reaction of ergotamine. The non-aromatic ring (ring *C* of the ergoline skeleton) directly fused to the aromatic rings is nearly planar [maximum deviation = 0.317 (4) Å] and shows an envelope conformation, whereas ring *D*, involved in an intra­molecular N—H⋯N hydrogen bond exhibits a slightly distorted chair conformation. The structure displays chains running approximately parallel to the diagonal of *bc* plane that are formed through N—H⋯O hydrogen bonds.

## Related literature

Ergotaminine is an ergot alkaloid formed by, among others, the fungus *Claviceps purpurea* on cereal grains and grasses during the growth process; see: Crews *et al.* (2009 ▶); Müller *et al.* (2009 ▶). For investigations of the biologically inactive C8-(*S*)-isomer ergotaminine, see: Pierri *et al.* (1982 ▶); Komarova & Tolkachev (2001 ▶). For the crystal structure of ergotamine tartrate ethanol solvate, see: Pakhomova *et al.* (1995 ▶). For the crystal structure of ergometrinine, another C8-(*S*)-configured ergotalkaloid, see: Merkel *et al.* (2010 ▶). For the solubility of ergotaminine, see: Stoll (1945 ▶).
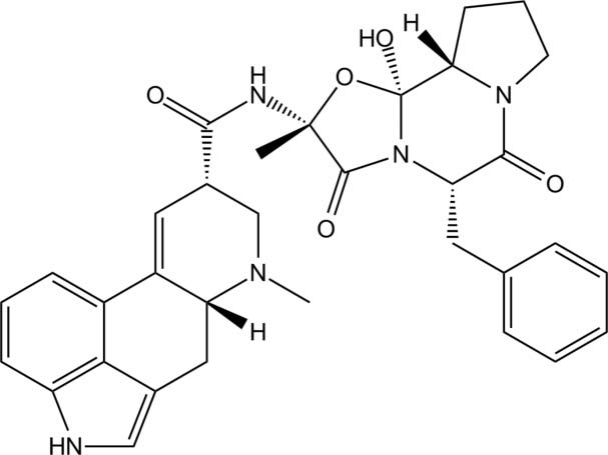


## Experimental

### 

#### Crystal data

C_33_H_35_N_5_O_5_
*M* *_r_* = 581.66
Monoclinic, 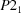

*a* = 10.974 (3) Å
*b* = 9.662 (2) Å
*c* = 14.450 (4) Å
β = 105.059 (15)°
*V* = 1479.5 (7) Å^3^
*Z* = 2
Mo *K*α radiation
μ = 0.09 mm^−1^
*T* = 296 K
0.2 × 0.1 × 0.06 mm

#### Data collection

Bruker APEX CCD area-detector diffractometer
Absorption correction: multi-scan (*SADABS*; Bruker, 2001 ▶) *T*_min_ = 0.879, *T*_max_ = 0.986
20196 measured reflections
2781 independent reflections
2240 reflections with *I* > 2σ(*I*)
*R*_int_ = 0.087

#### Refinement

*R*[*F* ^2^ > 2σ(*F* ^2^)] = 0.047
*wR*(*F* ^2^) = 0.093
*S* = 1.12
2781 reflections
390 parameters
1 restraint
H-atom parameters constrained
Δρ_max_ = 0.15 e Å^−3^
Δρ_min_ = −0.19 e Å^−3^
Absolute structure: determined from the synthesis

### 

Data collection: *SMART* (Bruker, 2001 ▶); cell refinement: *SAINT* (Bruker, 2001 ▶); data reduction: *SAINT*; program(s) used to solve structure: *SHELXS97* (Sheldrick, 2008 ▶); program(s) used to refine structure: *SHELXL97* (Sheldrick, 2008 ▶); molecular graphics: *SHELXTL* (Sheldrick, 2008 ▶) and *ORTEPIII* (Burnett & Johnson, 1996 ▶); software used to prepare material for publication: *SHELXTL*.

## Supplementary Material

Crystal structure: contains datablock(s) I, global. DOI: 10.1107/S1600536812003674/ds2173sup1.cif

Structure factors: contains datablock(s) I. DOI: 10.1107/S1600536812003674/ds2173Isup2.hkl

Supplementary material file. DOI: 10.1107/S1600536812003674/ds2173Isup3.mol

Additional supplementary materials:  crystallographic information; 3D view; checkCIF report

## Figures and Tables

**Table 1:** Hydrogen-bond geometry (Å, °)

*D*—H⋯*A*	*D*—H	H⋯*A*	*D*⋯*A*	*D*—H⋯*A*
N2—H2⋯N3	0.86	2.53	2.955 (4)	112
N4—H3⋯O5^i^	0.86	2.17	2.981 (5)	157

Symmetry code: (i) 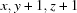.

**Fig. 1.**
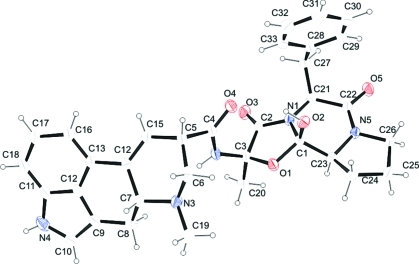
ORTEP representation of the title compound with atomic labeling shown with 30% probability displacement ellipsoids.

**Fig. 2.**
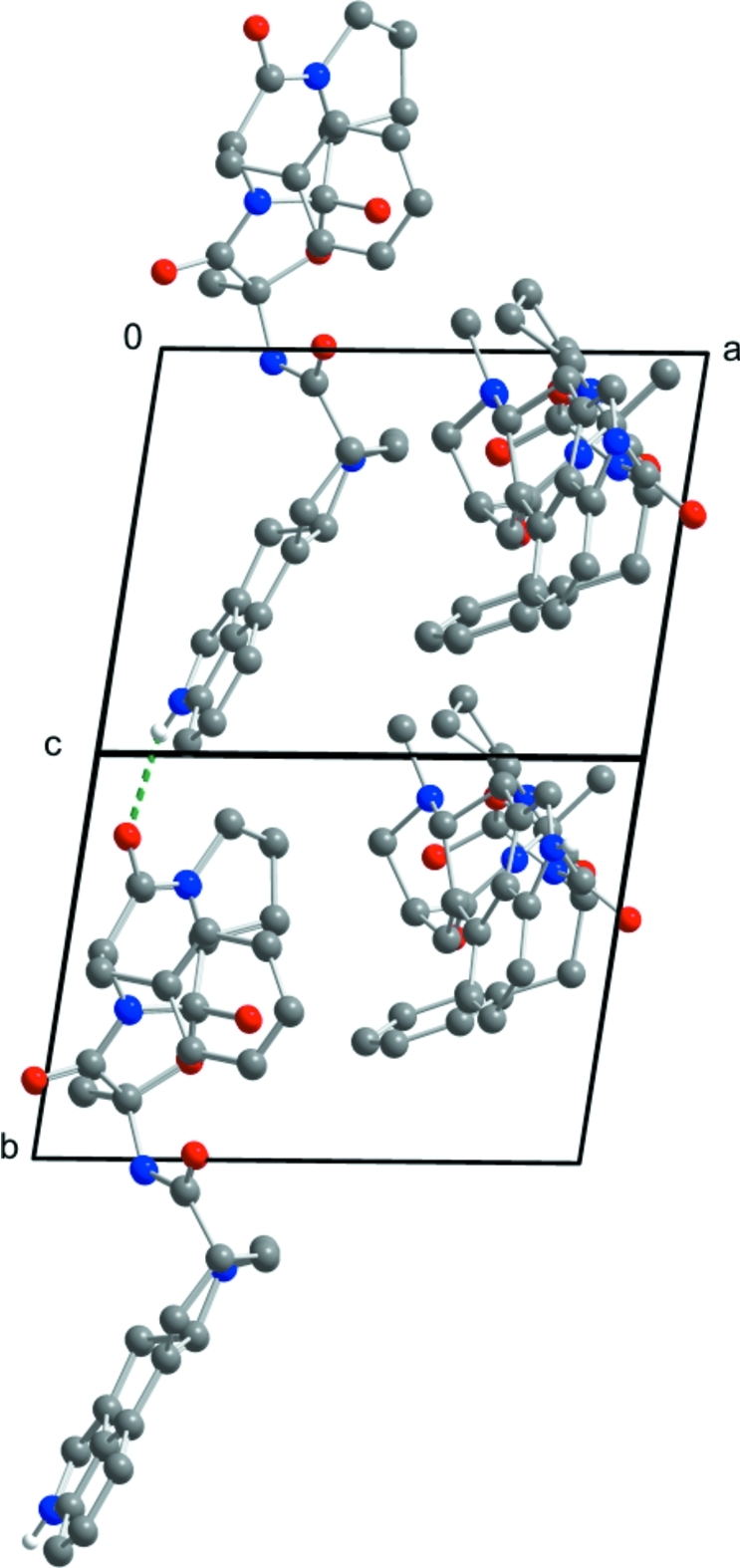
View of the unit cell of the title compound, showing the hydrogen-bonded chains are running approximately parallel to the diagonal of b-c plane. Hydrogen bonds are drawn as dashed green lines. H atoms are omitted for clarity.

## supplementary crystallographic information

### Comment

The fungus *Claviceps purpurea* is distributed worldwide through various climatic zones and produces a broad range of ergot alkaloids on grasses and cereal grains during the growth process whereas six epimeric pairs are predominantly formed. One of these main ergot alkaloids is ergotaminine. Contamination of flour and cereal based foods with ergot alkaloids including ergotaminine has previously been reported (Crews *et al.*, 2009; Müller *et al.*, 2009). The biologically inactive C8-(*S*)-isomer ergotaminine (Pierri *et al.*, 1982) can be converted to the biologically active C8-(*R*)-isomer ergotamine and *vice versa* (Komarova & Tolkachev, 2001). The molecule crystallizes in the monoclinic space group *P*2_1_. The molecular structure of the compound and the atom-labeling scheme are shown in Fig. 1. The absolute configuration could not be defined confidently based on the single-crystal diffraction data. It was however established based on liquid chromatography data that confirmed the epimeric purity of the obtained ergotaminine crystals. Besides the intramolecular hydrogen bonds between N2—H2 and N3 (see Table 1; not shown in Fig. 2), each molecule is connected to two adjacent molecules *via* intermolecular hydrogen bonds (see Table 1; see dashed green bonds in Fig. 2). As a result adjacent chains run along the [011] and [011] direction in an oppositely slanted fashion and with an inlined angle of 69.4°.

### Experimental

Ergotamine tartrate was obtained from Sigma–Aldrich (Taufkirchen, Germany). The stereoselective conversion of ergotamine to ergotaminine was carried out as follows: 12.4 mg ergotamine tartrate were dissolved in a solution of 5 ml methanol and 0.5 ml water. For epimerization reaction the resulting mixture was stored in a sealed vial in darkness at ambient temperature for two weeks. As a result of the slow crystallization colorless crystals of the title compound were formed, because of a substantial solubility difference between ergotamine and ergotaminine (as reported by Stoll (1945)). The isomeric purity (98%) of ergotaminine was proved by HPLC-FLD.

### Refinement

In the absence of significant anomalous dispersion effects, Friedel pairs were merged.

The N—H and O—H H atoms were located in difference maps and fixed in their found positions (AFIX 3) with *U*_iso_(H) = 1.2 of the parent atom *U*_eq_ or 1.5 *U*_eq_(C_methyl_, O).

### Crystal data

**Table:**

C_33_H_35_N_5_O_5_	*F*(000) = 616
*M**_r_* = 581.66	*D*_x_ = 1.306 Mg m^−^^3^
Monoclinic, *P*2_1_	Mo *K*α radiation, λ = 0.71073 Å
Hall symbol: P 2yb	Cell parameters from 86 reflections
*a* = 10.974 (3) Å	θ = 4–29°
*b* = 9.662 (2) Å	µ = 0.09 mm^−^^1^
*c* = 14.450 (4) Å	*T* = 296 K
β = 105.059 (15)°	Plate, colourless
*V* = 1479.5 (7) Å^3^	0.2 × 0.1 × 0.06 mm
*Z* = 2	

### Data collection

**Table:**

Bruker APEX CCD area-detector diffractometer	2781 independent reflections
Radiation source: fine-focus sealed tube	2240 reflections with *I* > 2σ(*I*)
Graphite monochromator	*R*_int_ = 0.087
ω/2θ scans	θ_max_ = 25.1°, θ_min_ = 1.5°
Absorption correction: multi-scan (*SADABS*; Bruker, 2001)	*h* = −12→12
*T*_min_ = 0.879, *T*_max_ = 0.986	*k* = −11→11
20196 measured reflections	*l* = −14→17

### Refinement

**Table:**

Refinement on *F*^2^	Secondary atom site location: difference Fourier map
Least-squares matrix: full	Hydrogen site location: inferred from neighbouring sites
*R*[*F*^2^ > 2σ(*F*^2^)] = 0.047	H-atom parameters constrained
*wR*(*F*^2^) = 0.093	*w* = 1/[σ^2^(*F*_o_^2^) + (0.0345*P*)^2^] where *P* = (*F*_o_^2^ + 2*F*_c_^2^)/3
*S* = 1.12	(Δ/σ)_max_ < 0.001
2781 reflections	Δρ_max_ = 0.15 e Å^−^^3^
390 parameters	Δρ_min_ = −0.19 e Å^−^^3^
1 restraint	Absolute structure: syn
Primary atom site location: structure-invariant direct methods	

### Special details

**Table:**

Geometry. All e.s.d.'s (except the e.s.d. in the dihedral angle between two l.s. planes) are estimated using the full covariance matrix. The cell e.s.d.'s are taken into account individually in the estimation of e.s.d.'s in distances, angles and torsion angles; correlations between e.s.d.'s in cell parameters are only used when they are defined by crystal symmetry. An approximate (isotropic) treatment of cell e.s.d.'s is used for estimating e.s.d.'s involving l.s. planes.
Refinement. Refinement of *F*^2^ against ALL reflections. The weighted *R*-factor *wR* and goodness of fit *S* are based on *F*^2^, conventional *R*-factors *R* are based on *F*, with *F* set to zero for negative *F*^2^. The threshold expression of *F*^2^ > σ(*F*^2^) is used only for calculating *R*-factors(gt) *etc*. and is not relevant to the choice of reflections for refinement. *R*-factors based on *F*^2^ are statistically about twice as large as those based on *F*, and *R*-factors based on ALL data will be even larger.

### Fractional atomic coordinates and isotropic or equivalent isotropic displacement parameters (Å^2^)

**Table:**

	*x*	*y*	*z*	*U*_iso_*/*U*_eq_	
O1	0.2567 (2)	0.1765 (2)	0.57682 (16)	0.0564 (7)	
O2	0.3529 (2)	0.2000 (2)	0.45142 (16)	0.0526 (6)	
H1	0.3525	0.2846	0.4559	0.079*	
O3	−0.0202 (3)	0.3535 (3)	0.45451 (19)	0.0753 (8)	
O4	0.2943 (3)	0.4688 (3)	0.52044 (19)	0.0787 (9)	
O5	0.0781 (3)	−0.0063 (3)	0.21039 (18)	0.0726 (8)	
N1	0.1306 (2)	0.2059 (3)	0.42571 (18)	0.0458 (7)	
N2	0.2054 (3)	0.3898 (3)	0.6360 (2)	0.0589 (8)	
H2	0.1851	0.4118	0.6877	0.071*	
N3	0.3774 (3)	0.4339 (3)	0.8282 (2)	0.0594 (8)	
N4	0.1372 (4)	0.7970 (5)	1.0691 (3)	0.0918 (13)	
H3	0.1068	0.8344	1.1123	0.110*	
N5	0.2018 (2)	−0.0396 (3)	0.36023 (19)	0.0478 (7)	
C1	0.2503 (3)	0.1457 (3)	0.4785 (2)	0.0438 (8)	
C2	0.0775 (3)	0.2859 (4)	0.4814 (3)	0.0529 (9)	
C3	0.1537 (3)	0.2655 (4)	0.5853 (2)	0.0513 (9)	
C4	0.2866 (4)	0.4728 (4)	0.6038 (3)	0.0606 (10)	
C5	0.3716 (4)	0.5658 (4)	0.6793 (3)	0.0626 (11)	
H4	0.4299	0.6130	0.6487	0.075*	
C6	0.4514 (4)	0.4756 (4)	0.7608 (3)	0.0710 (12)	
H5	0.4799	0.3936	0.7338	0.085*	
H6	0.5253	0.5270	0.7951	0.085*	
C7	0.3456 (3)	0.5530 (4)	0.8811 (3)	0.0563 (10)	
H7	0.4227	0.5813	0.9287	0.068*	
C8	0.2471 (4)	0.5103 (4)	0.9358 (3)	0.0649 (11)	
H8	0.1736	0.4707	0.8913	0.078*	
H9	0.2830	0.4407	0.9834	0.078*	
C9	0.2085 (4)	0.6349 (5)	0.9840 (3)	0.0637 (11)	
C10	0.1623 (4)	0.6591 (6)	1.0624 (3)	0.0858 (14)	
H10	0.1499	0.5914	1.1048	0.103*	
C11	0.1678 (4)	0.8663 (5)	0.9949 (3)	0.0732 (12)	
C12	0.2133 (3)	0.7655 (4)	0.9420 (3)	0.0573 (10)	
C13	0.2523 (3)	0.7954 (4)	0.8593 (3)	0.0532 (9)	
C14	0.3004 (3)	0.6764 (4)	0.8136 (2)	0.0487 (9)	
C15	0.3054 (3)	0.6754 (4)	0.7218 (3)	0.0548 (9)	
H11	0.2654	0.7467	0.6822	0.066*	
C16	0.2457 (4)	0.9325 (4)	0.8304 (3)	0.0661 (11)	
H12	0.2718	0.9580	0.7765	0.079*	
C17	0.1988 (4)	1.0347 (4)	0.8837 (3)	0.0831 (14)	
H13	0.1944	1.1262	0.8631	0.100*	
C18	0.1594 (4)	1.0026 (6)	0.9656 (3)	0.0841 (15)	
H14	0.1286	1.0705	0.9991	0.101*	
C19	0.4491 (5)	0.3282 (5)	0.8935 (3)	0.0904 (14)	
H15	0.4720	0.2545	0.8567	0.136*	
H16	0.3980	0.2920	0.9326	0.136*	
H17	0.5240	0.3690	0.9336	0.136*	
C20	0.0706 (4)	0.1934 (5)	0.6393 (3)	0.0718 (11)	
H18	0.0020	0.2532	0.6426	0.108*	
H19	0.1195	0.1718	0.7030	0.108*	
H20	0.0378	0.1095	0.6066	0.108*	
C21	0.0681 (3)	0.1685 (4)	0.3258 (2)	0.0502 (9)	
H21	−0.0208	0.1523	0.3235	0.060*	
C22	0.1174 (3)	0.0328 (4)	0.2951 (3)	0.0507 (9)	
C23	0.2471 (3)	−0.0094 (4)	0.4643 (2)	0.0470 (8)	
H22	0.1881	−0.0499	0.4973	0.056*	
C24	0.3714 (4)	−0.0875 (4)	0.4936 (3)	0.0651 (11)	
H23	0.4400	−0.0325	0.4822	0.078*	
H24	0.3914	−0.1134	0.5607	0.078*	
C25	0.3467 (4)	−0.2143 (4)	0.4293 (3)	0.0805 (13)	
H25	0.3059	−0.2862	0.4573	0.097*	
H26	0.4252	−0.2505	0.4202	0.097*	
C26	0.2613 (4)	−0.1660 (4)	0.3349 (3)	0.0577 (10)	
H27	0.3097	−0.1456	0.2891	0.069*	
H28	0.1984	−0.2355	0.3080	0.069*	
C27	0.0694 (4)	0.2867 (4)	0.2538 (3)	0.0658 (11)	
H29	0.0173	0.2584	0.1917	0.079*	
H30	0.0296	0.3669	0.2737	0.079*	
C28	0.1947 (4)	0.3310 (4)	0.2411 (2)	0.0551 (10)	
C29	0.2512 (5)	0.2625 (5)	0.1781 (3)	0.0706 (12)	
H31	0.2115	0.1859	0.1443	0.085*	
C30	0.3658 (5)	0.3069 (5)	0.1651 (3)	0.0863 (15)	
H32	0.4021	0.2597	0.1229	0.104*	
C31	0.4258 (5)	0.4192 (6)	0.2138 (4)	0.0921 (15)	
H33	0.5020	0.4494	0.2041	0.110*	
C32	0.3729 (5)	0.4871 (5)	0.2769 (3)	0.0867 (13)	
H34	0.4143	0.5624	0.3113	0.104*	
C33	0.2586 (4)	0.4445 (4)	0.2899 (3)	0.0667 (11)	
H35	0.2234	0.4927	0.3323	0.080*	

### Atomic displacement parameters (Å^2^)

**Table:**

	*U*^11^	*U*^22^	*U*^33^	*U*^12^	*U*^13^	*U*^23^
O1	0.0583 (15)	0.0628 (17)	0.0461 (14)	0.0085 (13)	0.0098 (12)	−0.0074 (12)
O2	0.0457 (13)	0.0436 (13)	0.0695 (15)	−0.0059 (11)	0.0167 (12)	0.0008 (12)
O3	0.0698 (18)	0.077 (2)	0.0795 (18)	0.0295 (17)	0.0199 (15)	−0.0057 (16)
O4	0.127 (3)	0.0594 (17)	0.0620 (17)	−0.0228 (17)	0.0460 (17)	−0.0118 (14)
O5	0.0844 (19)	0.0737 (18)	0.0501 (15)	0.0090 (16)	0.0004 (14)	−0.0174 (14)
N1	0.0429 (15)	0.0467 (16)	0.0466 (16)	0.0038 (14)	0.0095 (13)	−0.0067 (14)
N2	0.078 (2)	0.0510 (18)	0.0569 (19)	−0.0101 (16)	0.0347 (17)	−0.0146 (15)
N3	0.0624 (19)	0.0521 (19)	0.0624 (19)	0.0094 (16)	0.0140 (16)	−0.0056 (16)
N4	0.107 (3)	0.116 (4)	0.056 (2)	0.025 (3)	0.027 (2)	−0.015 (2)
N5	0.0486 (16)	0.0409 (16)	0.0509 (16)	0.0013 (13)	0.0077 (13)	−0.0074 (13)
C1	0.0418 (19)	0.0470 (19)	0.0424 (19)	−0.0020 (16)	0.0107 (16)	−0.0030 (15)
C2	0.053 (2)	0.046 (2)	0.062 (2)	0.0042 (19)	0.0190 (19)	−0.0042 (18)
C3	0.057 (2)	0.047 (2)	0.053 (2)	0.0019 (18)	0.0218 (18)	−0.0087 (17)
C4	0.080 (3)	0.051 (2)	0.058 (2)	−0.008 (2)	0.031 (2)	−0.013 (2)
C5	0.071 (3)	0.056 (2)	0.071 (3)	−0.014 (2)	0.037 (2)	−0.018 (2)
C6	0.059 (2)	0.065 (3)	0.093 (3)	0.000 (2)	0.026 (2)	−0.021 (3)
C7	0.054 (2)	0.056 (2)	0.054 (2)	0.0060 (19)	0.0054 (18)	−0.0090 (18)
C8	0.069 (3)	0.064 (3)	0.061 (2)	0.001 (2)	0.016 (2)	0.004 (2)
C9	0.061 (2)	0.082 (3)	0.047 (2)	0.008 (2)	0.012 (2)	−0.001 (2)
C10	0.095 (3)	0.109 (4)	0.054 (3)	0.013 (3)	0.022 (3)	0.004 (3)
C11	0.073 (3)	0.093 (4)	0.047 (2)	0.014 (3)	0.005 (2)	−0.017 (2)
C12	0.053 (2)	0.070 (3)	0.044 (2)	0.005 (2)	0.0044 (18)	−0.014 (2)
C13	0.051 (2)	0.053 (2)	0.051 (2)	0.0027 (18)	0.0046 (17)	−0.0153 (18)
C14	0.0437 (19)	0.052 (2)	0.049 (2)	−0.0028 (17)	0.0092 (16)	−0.0082 (17)
C15	0.060 (2)	0.048 (2)	0.058 (2)	−0.0043 (18)	0.0192 (19)	−0.0085 (18)
C16	0.071 (3)	0.064 (3)	0.060 (2)	0.005 (2)	0.013 (2)	−0.012 (2)
C17	0.100 (3)	0.059 (3)	0.081 (3)	0.017 (2)	0.007 (3)	−0.021 (2)
C18	0.094 (3)	0.094 (4)	0.056 (3)	0.025 (3)	0.005 (2)	−0.033 (3)
C19	0.091 (3)	0.070 (3)	0.100 (3)	0.023 (3)	0.006 (3)	0.006 (3)
C20	0.083 (3)	0.067 (3)	0.074 (3)	−0.011 (2)	0.036 (2)	−0.003 (2)
C21	0.0438 (19)	0.050 (2)	0.053 (2)	0.0042 (17)	0.0063 (16)	−0.0035 (17)
C22	0.047 (2)	0.051 (2)	0.052 (2)	0.0033 (17)	0.0077 (17)	−0.0039 (17)
C23	0.0468 (19)	0.0442 (18)	0.050 (2)	0.0003 (16)	0.0123 (16)	−0.0009 (16)
C24	0.068 (3)	0.049 (2)	0.070 (2)	0.009 (2)	0.003 (2)	0.001 (2)
C25	0.085 (3)	0.056 (2)	0.089 (3)	0.018 (2)	0.003 (2)	−0.012 (2)
C26	0.059 (2)	0.047 (2)	0.070 (2)	0.0059 (19)	0.021 (2)	−0.0106 (19)
C27	0.072 (3)	0.068 (3)	0.053 (2)	0.023 (2)	0.007 (2)	0.002 (2)
C28	0.067 (3)	0.050 (2)	0.046 (2)	0.0169 (19)	0.0103 (19)	0.0089 (18)
C29	0.093 (3)	0.065 (3)	0.058 (2)	0.014 (3)	0.028 (2)	−0.003 (2)
C30	0.116 (4)	0.075 (3)	0.081 (3)	0.034 (3)	0.049 (3)	0.013 (3)
C31	0.085 (3)	0.084 (4)	0.117 (4)	0.019 (3)	0.044 (3)	0.031 (3)
C32	0.091 (4)	0.070 (3)	0.094 (3)	0.001 (3)	0.015 (3)	0.003 (3)
C33	0.086 (3)	0.055 (3)	0.060 (2)	0.010 (2)	0.022 (2)	−0.001 (2)

### Geometric parameters (Å, º)

**Table:**

O1—C1	1.435 (4)	C13—C16	1.385 (5)
O1—C3	1.451 (4)	C13—C14	1.490 (5)
O2—C1	1.388 (4)	C14—C15	1.340 (5)
O2—H1	0.8201	C15—H11	0.9300
O3—C2	1.229 (4)	C16—C17	1.428 (6)
O4—C4	1.230 (4)	C16—H12	0.9300
O5—C22	1.246 (4)	C17—C18	1.396 (6)
N1—C2	1.353 (4)	C17—H13	0.9300
N1—C1	1.458 (4)	C18—H14	0.9300
N1—C21	1.474 (4)	C19—H15	0.9600
N2—C4	1.367 (5)	C19—H16	0.9600
N2—C3	1.444 (4)	C19—H17	0.9600
N2—H2	0.8600	C20—H18	0.9600
N3—C19	1.472 (5)	C20—H19	0.9600
N3—C7	1.472 (4)	C20—H20	0.9600
N3—C6	1.477 (5)	C21—C22	1.527 (5)
N4—C10	1.369 (6)	C21—C27	1.547 (5)
N4—C11	1.378 (6)	C21—H21	0.9800
N4—H3	0.8600	C23—C24	1.519 (5)
N5—C22	1.334 (4)	C23—H22	0.9800
N5—C26	1.475 (4)	C24—C25	1.519 (6)
N5—C23	1.485 (4)	C24—H23	0.9700
C1—C23	1.512 (5)	C24—H24	0.9700
C2—C3	1.529 (5)	C25—C26	1.514 (5)
C3—C20	1.515 (5)	C25—H25	0.9700
C4—C5	1.530 (5)	C25—H26	0.9700
C5—C15	1.503 (5)	C26—H27	0.9700
C5—C6	1.542 (6)	C26—H28	0.9700
C5—H4	0.9800	C27—C28	1.495 (5)
C6—H5	0.9700	C27—H29	0.9700
C6—H6	0.9700	C27—H30	0.9700
C7—C14	1.539 (5)	C28—C33	1.391 (5)
C7—C8	1.552 (5)	C28—C29	1.395 (5)
C7—H7	0.9800	C29—C30	1.387 (6)
C8—C9	1.506 (5)	C29—H31	0.9300
C8—H8	0.9700	C30—C31	1.366 (7)
C8—H9	0.9700	C30—H32	0.9300
C9—C10	1.376 (5)	C31—C32	1.370 (6)
C9—C12	1.407 (6)	C31—H33	0.9300
C10—H10	0.9300	C32—C33	1.378 (6)
C11—C18	1.379 (7)	C32—H34	0.9300
C11—C12	1.406 (5)	C33—H35	0.9300
C12—C13	1.400 (5)		
			
C1—O1—C3	111.6 (2)	C5—C15—H11	118.1
C1—O2—H1	109.5	C13—C16—C17	119.8 (4)
C2—N1—C1	112.7 (3)	C13—C16—H12	120.1
C2—N1—C21	124.1 (3)	C17—C16—H12	120.1
C1—N1—C21	122.8 (3)	C18—C17—C16	122.5 (4)
C4—N2—C3	121.4 (3)	C18—C17—H13	118.7
C4—N2—H2	119.3	C16—C17—H13	118.7
C3—N2—H2	119.4	C11—C18—C17	117.5 (4)
C19—N3—C7	111.6 (3)	C11—C18—H14	121.3
C19—N3—C6	108.5 (3)	C17—C18—H14	121.3
C7—N3—C6	112.0 (3)	N3—C19—H15	109.5
C10—N4—C11	108.9 (4)	N3—C19—H16	109.5
C10—N4—H3	125.7	H15—C19—H16	109.5
C11—N4—H3	125.5	N3—C19—H17	109.5
C22—N5—C26	122.0 (3)	H15—C19—H17	109.5
C22—N5—C23	126.8 (3)	H16—C19—H17	109.5
C26—N5—C23	111.1 (3)	C3—C20—H18	109.5
O2—C1—O1	111.5 (3)	C3—C20—H19	109.5
O2—C1—N1	112.8 (3)	H18—C20—H19	109.5
O1—C1—N1	104.0 (2)	C3—C20—H20	109.5
O2—C1—C23	109.2 (3)	H18—C20—H20	109.5
O1—C1—C23	109.5 (3)	H19—C20—H20	109.5
N1—C1—C23	109.8 (3)	N1—C21—C22	112.7 (3)
O3—C2—N1	126.2 (3)	N1—C21—C27	113.2 (3)
O3—C2—C3	126.1 (3)	C22—C21—C27	111.9 (3)
N1—C2—C3	107.5 (3)	N1—C21—H21	106.1
N2—C3—O1	108.9 (3)	C22—C21—H21	106.1
N2—C3—C20	109.3 (3)	C27—C21—H21	106.1
O1—C3—C20	110.9 (3)	O5—C22—N5	122.3 (3)
N2—C3—C2	115.7 (3)	O5—C22—C21	119.1 (3)
O1—C3—C2	103.4 (3)	N5—C22—C21	118.6 (3)
C20—C3—C2	108.5 (3)	N5—C23—C1	108.8 (3)
O4—C4—N2	122.1 (3)	N5—C23—C24	103.0 (3)
O4—C4—C5	122.2 (4)	C1—C23—C24	117.8 (3)
N2—C4—C5	115.7 (3)	N5—C23—H22	108.9
C15—C5—C4	115.8 (3)	C1—C23—H22	108.9
C15—C5—C6	109.0 (3)	C24—C23—H22	108.9
C4—C5—C6	109.4 (3)	C25—C24—C23	103.0 (3)
C15—C5—H4	107.4	C25—C24—H23	111.2
C4—C5—H4	107.4	C23—C24—H23	111.2
C6—C5—H4	107.4	C25—C24—H24	111.2
N3—C6—C5	110.9 (3)	C23—C24—H24	111.2
N3—C6—H5	109.5	H23—C24—H24	109.1
C5—C6—H5	109.5	C24—C25—C26	105.8 (3)
N3—C6—H6	109.5	C24—C25—H25	110.6
C5—C6—H6	109.5	C26—C25—H25	110.6
H5—C6—H6	108.0	C24—C25—H26	110.6
N3—C7—C14	110.9 (3)	C26—C25—H26	110.6
N3—C7—C8	110.3 (3)	H25—C25—H26	108.7
C14—C7—C8	112.1 (3)	N5—C26—C25	104.0 (3)
N3—C7—H7	107.8	N5—C26—H27	111.0
C14—C7—H7	107.8	C25—C26—H27	111.0
C8—C7—H7	107.8	N5—C26—H28	111.0
C9—C8—C7	109.9 (3)	C25—C26—H28	111.0
C9—C8—H8	109.7	H27—C26—H28	109.0
C7—C8—H8	109.7	C28—C27—C21	117.6 (3)
C9—C8—H9	109.7	C28—C27—H29	107.9
C7—C8—H9	109.7	C21—C27—H29	107.9
H8—C8—H9	108.2	C28—C27—H30	107.9
C10—C9—C12	105.4 (4)	C21—C27—H30	107.9
C10—C9—C8	136.4 (4)	H29—C27—H30	107.2
C12—C9—C8	118.1 (3)	C33—C28—C29	117.0 (4)
N4—C10—C9	110.3 (4)	C33—C28—C27	121.5 (4)
N4—C10—H10	124.8	C29—C28—C27	121.5 (4)
C9—C10—H10	124.8	C30—C29—C28	121.0 (4)
N4—C11—C18	133.8 (4)	C30—C29—H31	119.5
N4—C11—C12	106.2 (4)	C28—C29—H31	119.5
C18—C11—C12	120.0 (4)	C31—C30—C29	120.6 (5)
C13—C12—C11	123.4 (4)	C31—C30—H32	119.7
C13—C12—C9	127.4 (3)	C29—C30—H32	119.7
C11—C12—C9	109.2 (4)	C30—C31—C32	119.5 (5)
C16—C13—C12	116.8 (3)	C30—C31—H33	120.3
C16—C13—C14	127.0 (3)	C32—C31—H33	120.3
C12—C13—C14	116.2 (3)	C31—C32—C33	120.4 (5)
C15—C14—C13	123.6 (3)	C31—C32—H34	119.8
C15—C14—C7	122.2 (3)	C33—C32—H34	119.8
C13—C14—C7	114.2 (3)	C32—C33—C28	121.6 (4)
C14—C15—C5	123.9 (4)	C32—C33—H35	119.2
C14—C15—H11	118.1	C28—C33—H35	119.2
			
C3—O1—C1—O2	115.5 (3)	C9—C12—C13—C14	−3.5 (5)
C3—O1—C1—N1	−6.4 (3)	C16—C13—C14—C15	−23.1 (6)
C3—O1—C1—C23	−123.6 (3)	C12—C13—C14—C15	159.1 (3)
C2—N1—C1—O2	−111.3 (3)	C16—C13—C14—C7	156.5 (4)
C21—N1—C1—O2	75.9 (4)	C12—C13—C14—C7	−21.3 (4)
C2—N1—C1—O1	9.6 (4)	N3—C7—C14—C15	−7.1 (5)
C21—N1—C1—O1	−163.2 (3)	C8—C7—C14—C15	−130.9 (4)
C2—N1—C1—C23	126.7 (3)	N3—C7—C14—C13	173.2 (3)
C21—N1—C1—C23	−46.1 (4)	C8—C7—C14—C13	49.5 (4)
C1—N1—C2—O3	176.7 (4)	C13—C14—C15—C5	170.2 (3)
C21—N1—C2—O3	−10.6 (6)	C7—C14—C15—C5	−9.4 (5)
C1—N1—C2—C3	−8.9 (4)	C4—C5—C15—C14	113.1 (4)
C21—N1—C2—C3	163.8 (3)	C6—C5—C15—C14	−10.7 (5)
C4—N2—C3—O1	56.9 (4)	C12—C13—C16—C17	−1.0 (5)
C4—N2—C3—C20	178.3 (3)	C14—C13—C16—C17	−178.8 (4)
C4—N2—C3—C2	−59.0 (5)	C13—C16—C17—C18	0.5 (6)
C1—O1—C3—N2	−122.1 (3)	N4—C11—C18—C17	−178.9 (4)
C1—O1—C3—C20	117.6 (3)	C12—C11—C18—C17	−0.8 (7)
C1—O1—C3—C2	1.5 (4)	C16—C17—C18—C11	0.5 (7)
O3—C2—C3—N2	−62.1 (5)	C2—N1—C21—C22	−154.1 (3)
N1—C2—C3—N2	123.5 (3)	C1—N1—C21—C22	17.9 (4)
O3—C2—C3—O1	178.9 (4)	C2—N1—C21—C27	77.7 (4)
N1—C2—C3—O1	4.5 (4)	C1—N1—C21—C27	−110.3 (3)
O3—C2—C3—C20	61.1 (5)	C26—N5—C22—O5	4.5 (5)
N1—C2—C3—C20	−113.3 (3)	C23—N5—C22—O5	−174.5 (3)
C3—N2—C4—O4	19.6 (6)	C26—N5—C22—C21	−175.2 (3)
C3—N2—C4—C5	−157.6 (3)	C23—N5—C22—C21	5.7 (5)
O4—C4—C5—C15	117.7 (4)	N1—C21—C22—O5	−175.5 (3)
N2—C4—C5—C15	−65.1 (5)	C27—C21—C22—O5	−46.7 (4)
O4—C4—C5—C6	−118.7 (4)	N1—C21—C22—N5	4.2 (4)
N2—C4—C5—C6	58.5 (4)	C27—C21—C22—N5	133.1 (3)
C19—N3—C6—C5	169.6 (3)	C22—N5—C23—C1	−33.7 (4)
C7—N3—C6—C5	−66.7 (4)	C26—N5—C23—C1	147.2 (3)
C15—C5—C6—N3	47.1 (4)	C22—N5—C23—C24	−159.5 (3)
C4—C5—C6—N3	−80.4 (4)	C26—N5—C23—C24	21.4 (4)
C19—N3—C7—C14	166.1 (3)	O2—C1—C23—N5	−74.8 (3)
C6—N3—C7—C14	44.2 (4)	O1—C1—C23—N5	162.9 (2)
C19—N3—C7—C8	−69.1 (4)	N1—C1—C23—N5	49.3 (3)
C6—N3—C7—C8	169.0 (3)	O2—C1—C23—C24	41.9 (4)
N3—C7—C8—C9	−176.1 (3)	O1—C1—C23—C24	−80.4 (4)
C14—C7—C8—C9	−52.0 (4)	N1—C1—C23—C24	166.0 (3)
C7—C8—C9—C10	−154.0 (5)	N5—C23—C24—C25	−33.9 (4)
C7—C8—C9—C12	28.8 (5)	C1—C23—C24—C25	−153.6 (3)
C11—N4—C10—C9	−0.5 (6)	C23—C24—C25—C26	35.2 (4)
C12—C9—C10—N4	0.9 (5)	C22—N5—C26—C25	−178.9 (3)
C8—C9—C10—N4	−176.5 (4)	C23—N5—C26—C25	0.2 (4)
C10—N4—C11—C18	178.1 (5)	C24—C25—C26—N5	−22.0 (4)
C10—N4—C11—C12	−0.1 (5)	N1—C21—C27—C28	65.2 (4)
N4—C11—C12—C13	178.8 (3)	C22—C21—C27—C28	−63.3 (4)
C18—C11—C12—C13	0.2 (6)	C21—C27—C28—C33	−96.9 (4)
N4—C11—C12—C9	0.7 (5)	C21—C27—C28—C29	84.6 (4)
C18—C11—C12—C9	−177.8 (4)	C33—C28—C29—C30	−0.2 (5)
C10—C9—C12—C13	−179.0 (4)	C27—C28—C29—C30	178.3 (4)
C8—C9—C12—C13	−1.0 (6)	C28—C29—C30—C31	−0.1 (6)
C10—C9—C12—C11	−1.0 (4)	C29—C30—C31—C32	1.0 (7)
C8—C9—C12—C11	177.0 (3)	C30—C31—C32—C33	−1.4 (7)
C11—C12—C13—C16	0.7 (5)	C31—C32—C33—C28	1.1 (6)
C9—C12—C13—C16	178.4 (4)	C29—C28—C33—C32	−0.2 (5)
C11—C12—C13—C14	178.7 (3)	C27—C28—C33—C32	−178.7 (4)

### Hydrogen-bond geometry (Å, º)

**Table:**

*D*—H···*A*	*D*—H	H···*A*	*D*···*A*	*D*—H···*A*
N2—H2···N3	0.86	2.53	2.955 (4)	112
N4—H3···O5^i^	0.86	2.17	2.981 (5)	157

Symmetry code: (i) *x*, *y*+1, *z*+1.

## References

[bb1] Bruker (2001). *SMART*, *SAINT* and *SADABS* Bruker AXS Inc., Madison, Wisconsin, USA.

[bb2] Burnett, M. N. & Johnson, C. K. (1996). *ORTEPIII* Report ORNL-6895. Oak Ridge National Laboratory, Tennessee, USA.

[bb4] Crews, C., Anderson, W. A. C., Rees, G. & Krska, R. (2009). *Food Addit. Contam. Part B*, **2**, 79–85. 10.1080/02652030903042509 24784971

[bb5] Komarova, E. L. & Tolkachev, O. N. (2001). *Pharm. Chem. J.* **35**, 37–45.

[bb6] Merkel, S., Köppen, R., Koch, M., Emmerling, F. & Nehls, I. (2010). *Acta Cryst.* E**66**, o2275. 10.1107/S1600536810030825 PMC3008012 21588630

[bb7] Müller, C., Kemmlein, S., Klaffke, H., Krauthause, W., Preiss-Weigert, A. & Wittkowski, R. (2009). *Mol. Nutr. Food Res.* **53**, 500–507. 10.1002/mnfr.200800091 19360756

[bb8] Pakhomova, S., Ondráucek, J., Huusák, M., Kratochvíl, B., Jegorov, A. & Stuchlík, J. (1995). *Acta Cryst.* C**51**, 308–311.

[bb9] Pierri, L., Pitman, I. H., Rae, I. D., Winkler, D. A. & Andrews, P. R. (1982). *J. Med. Chem.* **25**, 937–942. 10.1021/jm00350a010 7120281

[bb10] Sheldrick, G. M. (2008). *Acta Cryst.* A**64**, 112–122. 10.1107/S0108767307043930 18156677

[bb11] Stoll, A. (1945). *Helv. Chim. Acta*, **28**, 1283–1308.

